# Mr. Steele, on the Torpidity of Swallows

**Published:** 1810-09

**Authors:** Andrew Steele

**Affiliations:** Writer to the Signet. Edinburgh


### *To the Editors of the Medical and Physical Journal.*

GENTLEMEN,

I Happened to read in the Medical and Physical Journal, for June 1810, a paper on the torpidity of animals, by Benjamin Smith Barton, M. D. Professor of Natural History at Philadelphia, extracted from the Philosophical Magazine, being remarks on an Essay on the same subject by Henry Reeve, M. D.

Dr. Barton writes as follows:—"There are two species of the genus *Hirundo* (Swallow) which are peculiarly disposed to pass the brumal season in the cavities of rocks, in the hollows of trees, and in other similar situations; where they have often been found in a *soporose* state. These species are the *Hirundo riparia*, or Sand Swallow, commonly called, in the united states, bank swallow and bank martin; and the *Hirundo Pelasgia*, or aculeated swallow, which we call chimney-bird and chimney-swallow. *There is no fact in Ornithology better established, than the fact of the occasional torpidity of these two species of Hirundo.* I say nothing of the torpidity of swallows under water. *But I do not wholly deny this fact.* And I take much pleasure in referring Dr. Reeve to a short paper, in the *Transactions of the American Philosophical Society*, vol. 6th, part first, relative to the discovery of a torpid swallow under a quantity of mud and leaves. The author of that paper was a most worthy and respectable man; and a man so religiously attached to truth, that I believe him to have been incapable of uttering a falsehood. He was, moreover, a man of nice observation, and of a philosophical turn of mind. Dr. Reeve asserts, that no swallows were ever found in all the rivers and lakes of England, Wales, Ireland, Scotland, or Switzerland, although fishermen are constantly employed on these their supposed hiding places; does he mean to say, that it has never been asserted by any of his countrymen, that swallows have been found torpid, under water, in *England*? Swallows are said to have been found torpid in the river Thames; and the fact seems to have been credited by some illustrious Englishmen in the 17th century; and among others, if I do not mistake, by the immortal William Harvey."

These animadversions of Dr. Barton have surprized me much; especially when he remarks, "I say nothing of the torpidity torpidity of swallows under water, but I do not *wholly* deny this fact."

Dr. Barton stands in an elevated situation; and, for the sake of science and truth, I cannot permit his paper to pass without observation. Dr. Barton told me, personally, that *several* house swallows were taken out of the water, from the bottom of a pond in America, *in presence of his father*, and by him restored to life.

Perhaps, through some channel, this paper of mine may reach Dr. Barton; and I hope he will be able to explain what to me appears a great inconsistency: that he should now say he does not *wholly* deny the fact of the torpidity of swallows under water, and refer to the solitary instance of an American recently giving an account of a single torpid swallow being discovered under a quantity of mud and leaves; when he formerly asserted that his own father was an eye-witness in America to *several* swallows being taken out of the bottom of a pond in a state of hibernation.

Dr. Barton and I were in 1787 members of the Natural History Society in the College of Edinburgh. On the 22d of November that year a debate took place in that Society on the torpidity of animals, and we were both engaged in this debate, in the course of which Dr. Barton, among a variety of other facts, stated what had passed in America in his father's presence, as to a number of swallows being found in winter on the letting out of the water from a mill pond.

I had remarked, that although many stories had been told of birds of this tribe diving before winter beneath the water into ponds and lakes, I accounted them fabulous, and only derived from vulgar or childish apprehension; because swallows may be seen in multitudes, in the course of their emigration, skimming along the surface of a pond, and soon after entirely disappear, it might hence childishly be supposed they had sunk themselves for the winter in the pond. But all this in my opinion was so inconsistent with every principle of the animal economy of these birds, that I would as soon believe a miracle in these days, of a man rising from the dead, as credit such a story as the torpidity of swallows under water during winter: yet what evidence, I added, have we for the whole matter of the hibernation of swallows but senile tales? And if I recollect rightly, the most of the members of the Natural History Society assented to this opinion. I am sure that many gentlemen then present, now eminent in different quarters of the world, were sceptical on every point that related to the torpidity of swallows during all winter in any situation whatever.

However,

However, when Dr. Barton stated what his father had seen and done, I was obliged to say, that what he averred was a fact of such a nature, that if given on the word and honour of a gentleman, and for precision committed to writing, I could not but admit it as conclusive.

Dr. Barton therefore, at my request, sat down and instantly at the table of the secretary, wrote what follows, and also at my desire signed it with his name.

"The common house swallow (Hirundo Domestica) has been found in great numbers in cleaning a mill-dam in Pennsylvania. It appears that the swallows have in many instances been seen perching on the bushes adjacent to the shore; have been seen to plunge into the water; from whence they have been taken out in a state of torpidity, almost the state of death; but by the gradual application of heat, many of them have recovered. *Several of these swallows have been taken out of the water in presence of my father, and by him were recovered to life. B. S. Barton.*"

This autograph of Dr. Barton is at this moment lying before me; and as an anonymous assertion from any one in a case of this nature would be unworthyof notice, or reply, I subscribe myself,

Yours, &c.

ANDREW STEELE,

WRITER TO THE SIGNET.

*Edinburgh, July* 22, 1810.

